# Effect of Heel Lift Insoles on Lower Extremity Muscle Activation and Joint Work during Barbell Squats

**DOI:** 10.3390/bioengineering9070301

**Published:** 2022-07-08

**Authors:** Zhenghui Lu, Xin Li, Rongrong Xuan, Yang Song, István Bíró, Minjun Liang, Yaodong Gu

**Affiliations:** 1 Faculty of Sports Science, Ningbo University, Ningbo 315211, China; luzhenghui_nbu@foxmail.com (Z.L.); 2011042028@nbu.edu.cn (X.L.); guyaodong@nbu.edu.cn (Y.G.); 2 The Affiliated Hospital of Medical School of Ningbo University, Ningbo 315020, China; 3 Faculty of Engineering, University of Szeged, 6720 Szeged, Hungary; nbusongyang@hotmail.com (Y.S.); biro-i@mk.u-szeged.hu (I.B.)

**Keywords:** barbell squat, OpenSim, lower limb biomechanics, muscle, joint work

## Abstract

The effect of heel elevation on the barbell squat remains controversial, and further exploration of muscle activity might help find additional evidence. Therefore, 20 healthy adult participants (10 males and 10 females) were recruited for this study to analyze the effects of heel height on lower extremity kinematics, kinetics, and muscle activity using the OpenSim individualized musculoskeletal model. One-way repeated measures ANOVA was used for statistical analysis. The results showed that when the heel was raised, the participant’s ankle dorsiflexion angle significantly decreased, and the percentage of ankle work was increased (*p* < 0.05). In addition, there was a significant increase in activation of the vastus lateralis, biceps femoris, and gastrocnemius muscles and a decrease in muscle activation of the anterior tibialis muscle (*p* < 0.05). An increase in knee moments and work done and a reduction in hip work were observed in male subjects (*p* < 0.05). In conclusion, heel raises affect lower extremity kinematics and kinetics during the barbell squat and alter the distribution of muscle activation and biomechanical loading of the joints in the lower extremity of participants to some extent, and there were gender differences in the results.

## 1. Introduction

The barbell squat is one of the most effective exercises for building lower extremity strength [1,2,3,4,5,6,7,8,9,10,11,12,13,14,15] and is widely used for strength training and rehabilitation. During barbell squat training or competition, athletes often wear a type of heel lift shoe or use other means to elevate the heel, which is thought to improve the range of motion (ROM) of the lower extremity joints and improve stability of movement during the deep squat [16,17,18,19,20,21]. However, there is still controversy about whether raising the heel will affect the barbell squat movement.

Previous studies have shown that elevating heel height while performing barbell squats reduces the ankle dorsiflexion angle and anterior trunk tilt [16,18,22,23,24], which might help reduce lumbar spine shear forces [25]. However, some studies have reported that heel elevation does not affect hip and knee kinematic parameters [6,23,24]. In a study by Lee et al. [6], there were no significant effects on trunk and knee kinematics, whether using an inclined platform or weightlifting shoes. The participants recruited in the study by Lee et al. were all recreational weight lifters [6], and their movement patterns when performing a high load (80% 1RM) barbell squat could have large individual differences. Moreover, in the study by Lee et al. [6], participants were asked to squat until their hips reached the level of their knees rather than reaching maximum depth, which may have resulted in some participants not having reached the full ROM that they could accomplish. Furthermore, it is worth noting that although the variability in results was not significant, Lee et al. still observed greater knee flexion angles when raising the heel. In a recently published review [11], Pangan et al. noted that most current studies have focused on the effects of heel elevation on the kinematics of the deep squat movement, and fewer studies have examined the effects on kinetics, which may provide some valuable evidence for these effects.

Although coaches and athletes have been hoping to improve the movement pattern of the barbell squat by elevating the heel, the human neuromuscular system has the specific ability to adapt and coordinate to changes in the external environment, and therefore, small heel changes might hardly have a significant effect on the movement. Perhaps, it is reasonable to speculate that in some studies [6], lifters with some experience already have a relatively regular movement pattern. When the heel is elevated, the trainer would actively adjust the joint activity to fit the change, thus, the kinematic effects of elevated heel height on the deep squat may be difficult to observe. This was also demonstrated in a study by Sayers et al. [25]. Therefore, it might be possible to provide additional insight by studying kinetic parameters and muscle activity.

During the barbell squat, it is often necessary to engage the joint with the optimal moment and angular velocity to perform the barbell squat better [26,27,28]. The output power is closely related to the coordination of forces [26], rather than a solely single muscle [29]. When the heel is raised, it might cause the angle and ROM of the lower extremity joints to change, which may modify the joint’s mechanical forces and affect the joint’s work. In addition, during the barbell squat, especially during descent, some lower limb muscles are actively engaged in eccentric contraction to maintain the stability of the movement, involving energy dissipation, which is essential for the control of the action. Currently, many studies have used energetic principles to investigate the work done by joints in various states of motion [30,31,32,33,34]. These studies often use line and bar graphs to produce reports of the work done by the joints, which may be difficult to interpret with a single overlapping line and error line [35]. Recently, Ebrahimi et al. proposed composite lower limb work (CLEW) [35], a visualization method for interpreting lower limb work that has been used for landing, jumping, and gait tasks [35,36,37]. However, little attention has been paid to the effect of heel height on lower limb work during the barbell squat. In conclusion, the effect of elevated heel height on the kinematics and kinetics of the barbell deep squat remains controversial due to the differences in study designs. Pangan et al. suggested [11] that relevant studies could consider the gender and analyze muscle activity, since joint mobility and anatomical differences between men and women may affect kinematics and kinetics [23,24], and exploring muscle activity may help to explain the test results.

As a multi-joint exercise, the barbell squat primarily requires activating the hip, knee, and ankle joints [11]. Several studies have used surface electromyography (EMG) to test the effects of different foot conditions on the barbell squat [23,25,38]. Anbarian et al. found that raising the heel reduced fatigue in the lower back muscles [39]. Johnston et al. found that raising the heel significantly increased muscle activation in the gastrocnemius [40]. However, it is difficult to obtain information about deeper muscles using surface EMG, and the fat between the skin and the muscle may affect the measurements. OpenSim is an open-source biomechanical modeling, simulation, and analysis software. It enables the calculation of muscle activation by minimizing the sum of squares of muscle activation, and by using simulations, it can be used for calculating the degree of activation of deep muscles.

Therefore, in this study, we used an OpenSim musculoskeletal model specifically for the deep squat to reveal the effects of insoles with different heel heights on lower extremity muscle activation and work done during the barbell deep squat [8]. Based on previous studies, we hypothesized that an elevated heel would reduce mobility, extension moments, and joint work reduction in the hip and ankle joints instead of the knee.

## 2. Materials and Methods

### 2.1. Participants

The sample size for the within-factor repeated measures ANOVA (1 group × 3 measurements) was calculated using G*Power 3.1 (Franz Faul, Germany), as suggested by Faul et al. [41], taking into consideration Cohen’s medium effect size (d = 0.5), α error probability = 0.05, and power (1 − β) = 0.8. Based on these parameters, it was estimated that a minimum of 9 participants would be required for this study.

Twenty participants (female/male, 10/10) were eventually recruited for the experiment. All the participants were healthy and had more than 12 months of strength training experience, and the 1RM load of the barbell squat was between 1 and 1.2 times body weight for the female participants and between 1.75 and 2 times body weight for the male participants. They had no history of back and lower limb pain or injury in the most recent year and did not have any exercise in the 72 h before the experiment. All participants were aware of the test’s purpose, method, and steps and filled in the informed consent form. The study was approved by the Ethics Committee of Ningbo University. Table 1 summarizes the demographic information related to the age, height, and weight of the participants.

### 2.2. Experimental Design

**Figure 1:**
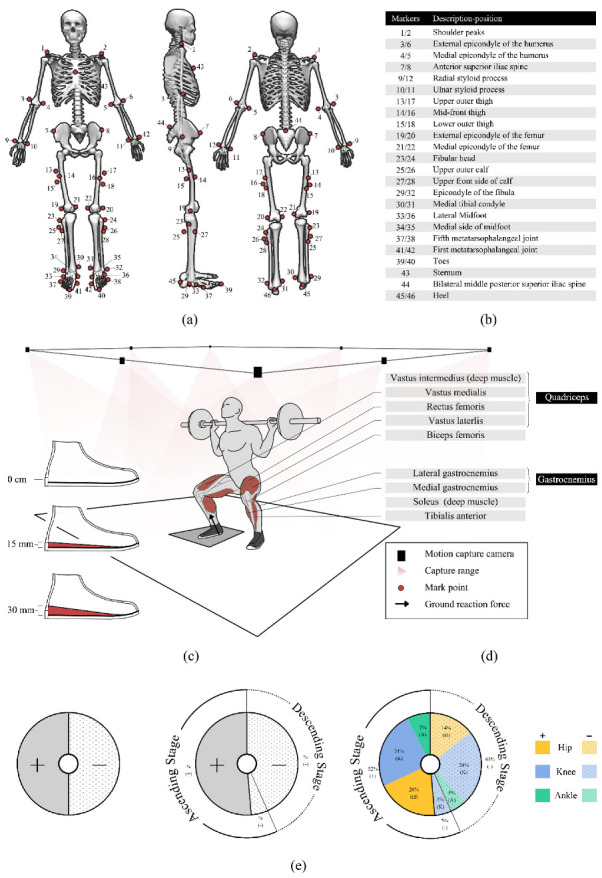
(**a**) Marking point paste location; (**b**) description of marker point location; (**c**) lab environment, barbell squatting movements, major muscle groups, and heel life insoles; (**d**) major muscle descriptions and illustrations; (**e**) Creation of a CLEW method pie chart for barbell squats; (**e-left**) first, the pie chart is divided according to the positive and negative work done. Starting from the centerline, the positive work is on the left side of the circle (dark), while the negative work is on the right side of the process (light dot); (**e-middle**) secondly, the pie chart is divided according to the descending and ascending stages of the squatting movement. The dotted part of the outer circle indicates the descending phase of the squatting movement, and the solid line represents the ascending stage. The percentage of the relative positive or negative work in the absolute stage work is expressed in the outer ring; (**e-right**) finally, each relevant constituent work is designated as part of the pie chart, squatting down in the order of the hips, knees, and ankles, starting with the central reference line, and the colors and patterns inside the pie chart are used to distinguish joints and to separate positive work from negative work.

Reflective point trajectories were captured using a Vicon 3D motion capture system (v. 1.8.5, Vicon Motion System, Oxford, UK) with eight cameras (MX-T-Series) at 200 Hz and an AMTI 3D force meter (AMTI, Watertown, MA, USA) at 1000 Hz to capture the marker point trajectory and ground reaction force simultaneously (Figure 1c,d). At least two cameras could capture the reflective markers during the entire barbell squat movement, avoiding gaps during data collection or marker points in a particular direction obscuring the barbell, therefore, ensuring accuracy.

The surface hair, body surface grease, sweat, and other foreign matter that may affect EMG signal collection were removed with a razor before attaching the sensor. The sensor was attached to the muscle belly of the target muscle, and the electrodes were placed parallel to the direction of the muscle fibers, with the direction of the muscle fibers referenced to the bony markers on the participant’s body surface. Using the rectus femoris muscle as an example, the electrodes need to be placed at 50% on the line from the anterior spina iliaca superior to the superior part of the patella. The EMG signals of the rectus femoris, biceps femoris longus, tibialis anterior muscle, and gastrocnemius muscle were collected synchronously with 1000 Hz frequency using a wireless Delsys EMG test system (Delsys, Boston, MA, USA) for model verification. The maximum voluntary contraction (MVC) was measured using a CON-TREX dynamometer (CON-TREX MJ System, CMV, Dübendorf, Switzerland). The original EMG signal was first filtered by bandpass fourth-order Butterworth filter in the frequency range of 100–500 Hz in Delsys EMG works analysis software. The amplitude analysis is carried out using root mean square (RMS) calculation. The MVC and standardized activity value of each action was output. The EMG activity was calculated from 0 (0, completely inactive) to 1 (100%, fully activated) through the test root mean square amplitude/MVC root mean square amplitude.

All participants performed a deep squat weight limit test under the guidance of a relevant practitioner. Participants performed a 5 min squat exercise to warm up before the experiment. In this study, only the dominant foot of the participants was analyzed. The dominant foot was determined using the customary soccer kick. All participants involved in the experiment had the right side as the dominant foot. All participants wore uniformly provided tights during the test, and the marking points were taped by the same skilled tester (Figure 1a,b). The calibration procedure of the VICON 3D motion capture system was performed according to the recommendations of the camera manufacturer. Each participant performed barbell squats in a randomized order wearing shoes with 0 cm, 1.5 cm, and 3 cm heel inserts (Figure 1c). The participants could not be blinded because the participants perceived the intervention conditions. Each participant used a high bar position squat with a weight selection of 70% 1RM and the right foot in contact with the force platform during the test. The 70% 1RM load is considered to be a typical choice of training load [23] and is also considered to have low movement variability and high retest reliability [42,43,44,45] to maintain the level of repeatability required to obtain a representative dataset. The test began with the knees in an upright position, squatting down to the deepest point while ensuring a neutral pelvic position and keeping the heels off the ground. Then, participants extended the hips, knees, and ankles, and returned to the upright position and the end of the movement. There was at least 2 min between each deep squat, and then participants rated their perceived exertion using a scale to ensure participants were well rested. All variables (min, max, and ROM) were obtained at each squat, and then averaged for each group. Finally, three sets of data for each heel height condition were used for statistical purposes.

### 2.3. Musculoskeletal Model and Model Validation

A customized deep squat model was used in this study [8,46]. This model has been used several times in previous studies [8,47]. The model was validated using the standardized muscle electrical signals obtained from the Delsys EMG test system measurements as compared with the degree of muscle activation output by the OpenSim static optimization algorithm. In addition, joint angles and joint moments were compared to data obtained in other studies to verify the accuracy of the model.

### 2.4. Data Processing

The data were processed using Vicon Nexus (v.1.8.5, Vicon Motion system, Oxford, UK). The trajectory data of the marker points and ground reaction forces were filtered using 12 Hz and 60 Hz [8]. The filtering was performed by using a personalized MATLAB program (v2017a, The MathWorks Inc., Natick, Middlesex, MA, USA). Then, the trajectory and ground reaction force data were converted and processed using MATLAB (such that the c3d format files were converted to trc and mot formats usable for OpenSim input).

The weights of the marker points in the model were manually adjusted, and the model was scaled to match the anthropometric characteristics of the participants so that the root mean square error of the marker points and virtual marker points in the experiment was less than 0.02, and the maximum error was less than 0.04. Then, the joint angles and moments were calculated using the inverse kinematics and inverse dynamics algorithm tools in OpenSim. Static optimization was used to estimate the degree of muscle activation during exercise for the major muscle groups, including the rectus femoris, vastus medialis, vastus lateralis, vastus intermedius, biceps femoris, gastrocnemius, soleus, and tibialis anterior.

Hip and knee extension and metatarsal flexion of the ankle joint were defined as positive numbers. Joint power was calculated as the product of the joint moment and joint angular velocity (determined from kinematic data) [26]. Mechanical work was calculated as the joint power accumulated over time, with negative work values indicating the energy dissipation of the muscle due to eccentric contraction (e.g., the quadriceps muscle is elongated during knee flexion when the quadriceps is in an eccentric contraction state) [26,48,49,50,51]. Participants’ power and mechanical work were normalized based on the sum of the participant’s body weights and the weight of the barbell used.

In the present study, the work done by the limb was visualized with reference to the CLEW method proposed by Anahid et al. [35]. Absolute work was defined as the sum of positive and negative work done by the limbs (hip, knee, and ankle) throughout the squat cycle. Relative work was defined as the absolute value of the work done by each component as a percentage of the absolute total. The pie chart for the CLEW method in this study was created using the steps described in (Figure 1e).

### 2.5. Statistical Analysis

Normality and sphericity hypotheses were verified using Shapiro–Wilk’s and Mauchly’s tests, respectively. Means and standard deviations were calculated for kinematics, kinetics, and degree of muscle activation for each heel height condition. One-way repeated measures ANOVAs were performed using IBM SPSS Statistics (version 26, SPSS AG, Zurich, Switzerland), with Bonferroni’s post hoc tests performed when necessary. If the sphericity assumption was violated, the degrees of freedom were adjusted using Greenhouse–Geisser correction. In addition, effect sizes were calculated using partial eta² ($\eta_{p}^{2}$), with the relative magnitude of any differences expressed as a standard criterion: small effect size = 0.01, medium effect size = 0.06, and large effect size = 0.14 [52]. Significance was set at *p* < 0.05.

## 3. Results

### 3.1. Model Validation

**Figure 2:**
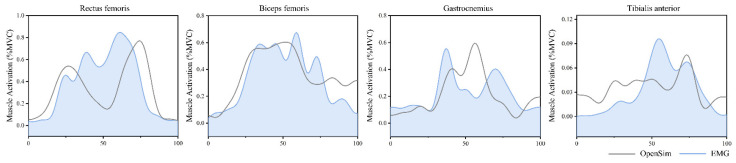
The results of rectus femoris, gastrocnemius, long biceps femoris, and anterior tibialis activation levels obtained using the EMG signal and the OpenSim optimization algorithm were more consistent.

As shown in Figure 2, the muscle activation calculated using the OpenSim static optimization tool during barbell squatting is similar to the EMG signal activity recorded in the experiment, indicating that the data of the OpenSim model in this study is reliable [53,54]. The joint angle and moment obtained by the OpenSim inverse kinematics and inverse kinetics algorithm are similar to previous studies [8,23,47].

### 3.2. Joint Angle, Joint Moment, and Joint Power

In Table 2, the results show no significant differences in joint angle, joint moment, and power of the hip joint regardless of the height of the insoles used (*p* > 0.05). Only male participants showed a significant increase in peak knee extension moment (*p* < 0.001, F = 19.434, $\eta_{p}^{2}$ = 0.599) and a significant increase in mean eccentric power (*p* = 0.009, F = 5.745, $\eta_{p}^{2}$ = 0.307) was observed when the heel was elevated using the 3 cm insole as compared with the 0 cm and 1.5 cm insoles. With increasing heel height, a significant main effect was observed both in the male group (*p* = 0.001, F = 10.870, $\eta_{p}^{2}$ = 0.455) and the female group (*p* < 0.001, F = 84.258, $\eta_{p}^{2}$ = 0.849), as well as the combined data from both genders (*p* < 0.001, F = 56.894, $\eta_{p}^{2}$ = 0.662); a significant main effect of maximum plantarflexion angle of the ankle was observed. In all participants (*p* < 0.001, F = 28.023, $\eta_{p}^{2}$ = 0.491) and in the female group (*p* < 0.001, F = 51.878, $\eta_{p}^{2}$ = 0.776), the maximum ankle dorsiflexion angle significantly decreased as the heel was elevated. In addition, the combined data from both genders showed greater ankle ROM when using 3 cm versus 1.5 cm insoles (*p* = 0.047, F = 3.231, $\eta_{p}^{2}$ = 0.116); when using 1.5 cm insoles, the ankle ROM of female subjects was significantly smaller than when using 0 cm insoles (*p* = 0.011, F = 5.316, $\eta_{p}^{2}$ = 0.262); when using 3 cm insoles, male subjects had significantly greater ankle ROM than when using 0 cm insoles (*p* = 0.014, F = 5.087, $\eta_{p}^{2}$ = 0.281).

### 3.3. Joint Work

**Figure 3:**
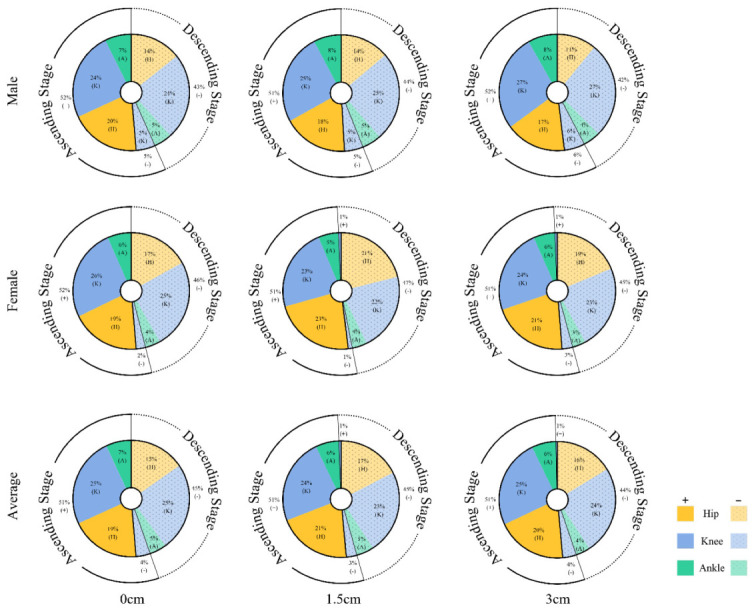
The percentage of work done by the hip, knee, and ankle joints during the descent and ascent of the barbell squat for different genders and heel heights. The percentage of work done by the joints was obtained by processing the joint power data.

The proportion of relative work done by the joints during squatting is shown in Table 3 and Figure 3. The combined data from both genders showed that, when using the 3 cm insole, the percentage of positive work (*p* < 0.001, F = 103.365, $\eta_{p}^{2}$ = 0.781) and negative work (*p* < 0.001, F = 17.008, $\eta_{p}^{2}$ = 0.370) performed by the ankle joint were significantly greater than when using 0 cm and 1.5 cm insoles. In female subjects, the percentage of positive (*p* < 0.001, F = 67.250, $\eta_{p}^{2}$ = 0.818) and negative (*p* = 0.001, F = 14.721, $\eta_{p}^{2}$ = 0.495) work performed on the ankle joint was significantly greater with the 3 cm insole than with the 0 cm and 1.5 cm insoles. In male subjects, the negative work performed on the knee joint was significantly greater with the 3 cm heel height insole than with the 0 cm insole (*p* = 0.040, F = 3.032, $\eta_{p}^{2}$ = 0.103). In addition, a significant main effect was observed in male subjects with higher insoles for both positive (*p* = 0.005, F = 6.699, $\eta_{p}^{2}$ = 0.34) and negative (*p* = 0.006, F = 6.281, $\eta_{p}^{2}$ = 0.344) work done by the hip joint and the percentage of positive work done by the ankle joint (*p <* 0.001, F = 44.394, $\eta_{p}^{2}$ = 0.773) both showed a significant main effect, with the percentage of work done by the hip decreasing and the ankle increasing with greater heel height.

### 3.4. Muscle Activation

**Figure 4:**
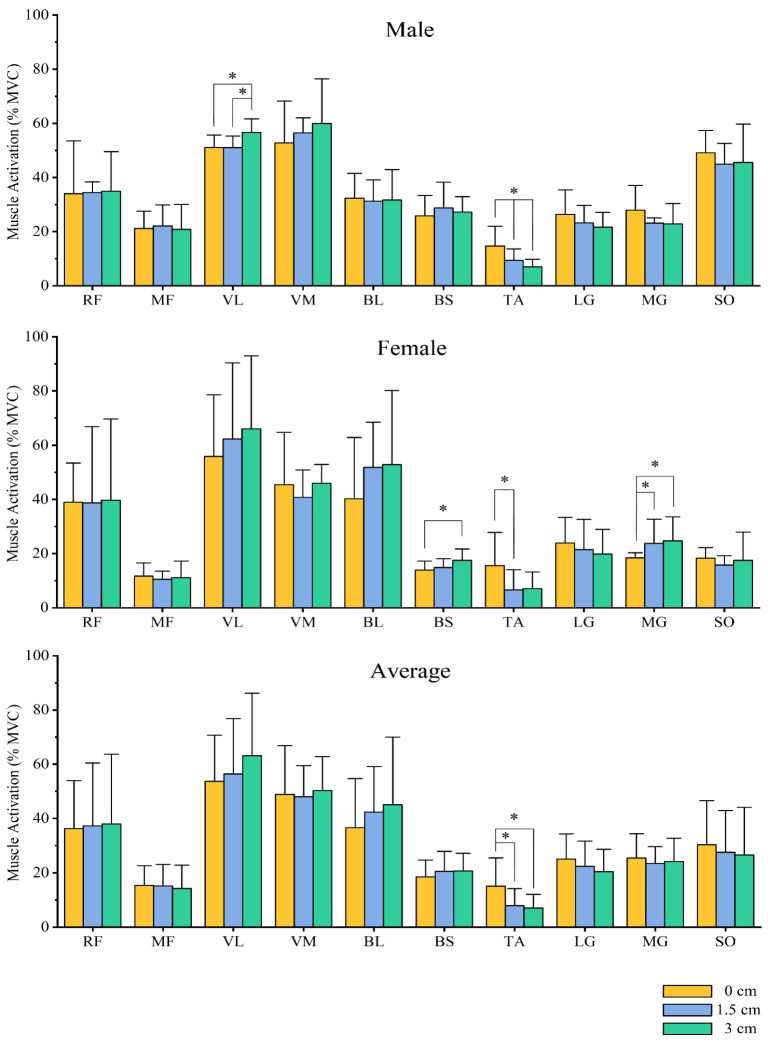
Mean standardized muscle activation during the barbell squat in males, females, and all participants. * Significant differences between the two groups. (RF, rectus femoris; MF, middle femoral; VL, vastus lateralis; VM, vastus medialis; BL, biceps femoris long head; BS, biceps femoris short head; TA, tibialis anterior; LG, lateral gastrocnemius; MG, medial gastrocnemius; SO, soleus). The OpenSim static optimization algorithm obtained the muscle activation data.

The average degree of muscle activation during squatting is shown in Figure 4. In male subjects, there was a significant main effect of mean muscle activation in the vastus lateralis (*p* < 0.001, F = 10.870, $\eta_{p}^{2}$ = 0.364) and anterior tibialis (*p* < 0.001, F = 11.854, $\eta_{p}^{2}$ = 0.384) during deep squatting with different insole heights. Post hoc pairwise comparisons showed that the mean muscle activation of the vastus lateralis was significantly greater in male subjects with the 3 cm insole than with the 0 cm and 1.5 cm insoles. The average muscle activation of the anterior tibialis muscle decreased significantly with increasing insole height.

In female subjects, there was a significant main effect of mean muscle activation in the short head of the biceps femoris (*p* = 0.003, F = 6.651, $\eta_{p}^{2}$ = 0.259), anterior tibialis (*p* = 0.01, F = 5.183, $\eta_{p}^{2}$ = 0.214), and medial gastrocnemius (*p* = 0.018, F = 4.508, $\eta_{p}^{2}$ = 0.192) when using different insole heights. Post hoc pairwise comparisons showed that the mean muscle activation of the short head of the biceps femoris was significantly greater in female subjects during the barbell squat when using a 3 cm insole than when using a 0 cm insole. When using a 1.5 cm insole, the average muscle activation of the anterior tibialis was significantly less than when using a 0 cm insole. With an increase in the heel height, the average muscle activation of the medial gastrocnemius increased; the average muscle activation of the medial gastrocnemius increased when using a 1.5 cm insole. The combined data from both genders showed that the mean muscle activation of the anterior tibialis showed a significant main effect (*p* = 0.01, F = 6.082, $\eta_{p}^{2}$ = 0.403) when using different height insoles, and the mean muscle activation of the medial gastrocnemius increased when using 1.5 cm and 3 cm insoles. The mean muscle activation of the anterior tibialis was significantly greater with 1.5 cm and 3 cm insoles than with 0 cm insoles.

## 4. Discussion

Barbell squats are commonly used for strength training and rehabilitation [1,2,3,4,5,6,7,8,9,10,11,12,13,14,15]. Heel elevation during a barbell squat is thought to improve deep squat movement patterns. However, current research on the effects of heel elevation on the deep squat has focused on kinematics, with little evidence on aspects such as kinetics and muscle activity. Therefore, in this study, we used the OpenSim musculoskeletal model customized for the deep squat to compare the effects of different heels on joint angles, joint moments, joint power, joint work done, and the degree of muscle activation during the barbell deep squat.

### 4.1. Joint Angle, Joint Moment, and Power

Participants’ ankle angles were directly affected during the barbell squat when heel height was changed. In the present study, it was observed that as heel height increased, participants exhibited progressively greater ankle plantarflexion and progressively less ankle dorsiflexion during the barbell squat, which was consistent with our hypothesis. When the heel is raised, the ankle is forced into a higher degree of plantarflexion, decreasing the ankle dorsiflexion angle [12,18,23,55,56], which may indirectly affect the knee or trunk angle [11]. In contrast, Charlton et al. showed that participants’ ankle dorsiflexion angles did not change with heel height [57]. This difference in results may be due to the experimental design. In the study by Charlton et al., participants used a uniform weight of 20 kg, which was well below the participants’ regular weights in training. While smaller weights may allow participants to squat with minimal fatigue or technical bias [57], higher weights may have other effects on movement [15], therefore, the results by Charlton et al. might not fully explain the effect of heel height. In conclusion, individuals with low ankle ROM often find it difficult to perform large dorsiflexion movements, therefore, there might be potential benefits to using heel elevation insoles during the barbell squat.

Similar to the ankle, the kinematics of the knee joint are very easily observed during the barbell deep squat. Raising the heel has been observed to increase knee ROM and extension moment in many previous studies [18,20,24,57], similar to the results obtained in this study. However, in this study, raising the heel height was observed to increase the moment and mean eccentric power only in male participants, but did not cause a significant change in knee mobility, which was partially consistent with our hypothesis. The differences in experimental design may explain the conflicting results in knee angles, such as whether the control group wore the same style of footwear, whether the depth of the squat had been maximized, and individual differences in squatting habits. In this study, participants were all wearing uniform footwear to control these factors and received instruction from the same coach not associated with the study. In a recent study [17], Monteiro et al. observed that the ROM of the knee joint increased with heel elevation during the barbell squat. Still, the difference in knee mobility was only statistically significant when the heel was elevated by 5 cm. Although the knee kinematics were not statistically significant in this study, there was still a tendency for peak knee flexion and mobility to be greater with an increase in heel height in all three cases. Therefore, elevating the heel may still have a potential effect on knee joint angle during the barbell squat, which is consistent with the findings of Monteiro et al. In addition, however, a significant main effect of knee moment was observed only in male participants, and there was a gradually increasing trend in knee moment and in power as the heel height increased. The results of biomechanical testing usually need to be analyzed in conjunction with the purpose of the intervention. This study suggests that heel elevation may increase knee extension moment and power during deep squats, implying that the knee joint might be subjected to greater loading. Still, this change in movement pattern is significant for individuals seeking strength, muscle hypertrophy, and rehabilitation; therefore, a moderate increase in heel height may enhance the knee joint training effect. The differences in experimental design may explain the conflicting results in knee angles, such as whether the control group wore the same style of footwear, whether the depth of the squat had been maximized, and individual differences in squatting habits. In this study, participants were all wearing uniform footwear to control these factors and received instruction from the same trainer not associated with the study. In a recent study [17], Monteiro et al. observed that knee ROM increased with heel elevation during the barbell squat. Still, a significant difference was only shown when the heel was elevated by 5 cm. Similar to the findings of Monteiro et al., although knee kinematics did not show significant differences in our study, there was still a tendency for peak knee flexion and mobility to be greater with an increase in heel height. Therefore, heel elevation may still have a potential effect on knee angulation. Furthermore, although a significant main effect of knee moment was only observed in male participants, all participants also showed a gradual increase in knee moment and in power with increasing heel height. In conclusion, heel elevation during the deep squat may increase knee extension moment and power, but it also means that the knee joint may be subjected to greater loading. Nevertheless, this change in movement pattern is important for those seeking strength, muscle hypertrophy, and rehabilitation; therefore, a moderate increase in heel height may enhance the knee joint training effect.

In this study, no statistically significant differences in hip joint angle, joint moment, or power were found regardless of the height of the insole used by the participants in the barbell squat. Although it is widely believed that elevating the heel during the barbell squat might result in a more upright trunk to prevent back injury, the current findings suggest that heel height only has little effect on hip flexion angle [20,23,24,58] and no statistically significant effect on hip and lower back muscle activation was found [58]. Notably, although raising the heel had little effect on the hip flexion angle during the barbell squat, the tilt angle of the trunk decreased with heel elevation in the study by Monteiro et al. [17]. This may result in a biomechanical transfer from the hip joint to the knee joint, contributing to the increased knee moment.

### 4.2. Joint Work

A comparative analysis of the proportion of lower extremity joints doing work showed that the proportion of hip joint work decreased with increasing heel height. In contrast, the proportion of ankle joint work increased gradually. Combined with the results of other indices, it can be hypothesized that when heel height is increased, male participants prefer to use the knee joint rather than the hip joint to lift the barbell. In contrast, the ankle joint needs to improve plantarflexion performance to enhance trunk stability. Although an increase in ankle work ratio was also observed in female participants, the knee work ratio did not change significantly with the increased heel height. In contrast, the hip work ratio increased with a 1.5 cm heel, which may be influenced by the physiological differences between gender and the level of training. To the best of our knowledge, this is the first attempt to analyze the effect of heel height on the lower extremity joint work ratio. Therefore, the current evidence could not draw definitive conclusions about the specific effects of heel height on lower extremity joint work during the barbell squat, and more evidence would be helpful.

### 4.3. Muscle Activation

This study suggests that heel elevation may increase activation of the knee extensor and the metatarsal flexor muscle group, similar to the results of previous studies [40,59]. In a study by Christopher et al. [40], although the results were not statistically significant, there was a slight increase in muscle activation of the lateral vastus and medial vastus muscles with a raised heel, and the average muscle activation of the gastrocnemius was significantly higher when raising the heel [40,59], which was similar to our results. This might be the result of the raised heel causing the movement of the body’s center of mass and the center of pressure, which forces participants to activate the metatarsal flexor muscle group to control balance and resist the body’s center of mass forward during the squat [11,40]. In several previous studies, differences in muscle activation only reached statistically significant levels when the heel was raised high enough [40,59]. In this study, the mean muscle activation of the dorsiflexion and plantarflexion muscle groups was significantly altered when the heel was raised by 1.5 cm, and the mean activation of the vastus lateralis and biceps femoris short head was increased considerably when raised by 3 cm. These results are essential for both training and rehabilitation. For athletes who want to improve the strength of the knee joint muscles, heel elevation can be used to increase the activation of the knee muscles to improve training results, and the activation of the ankle flexors and extensors can be an important reference for developing ankle stability training.

### 4.4. Limitations

This study still has some limitations. The main results are as follows: (1) The study of kinematics and kinetics are only considered in the sagittal plane. (2) Although the participants were all enthusiasts who had undergone strength training for more than one year, the maximum weight of barbell squats in males was no more than two times their body weight, and that in women was about one times their body weight. However, it is impossible to know whether the results of this study apply to higher level lifters.

## 5. Conclusions

This study showed that although some of the statistical differences were minor, raising the heel might still affect the joint ROM, joint moments, and muscle activation in the lower extremity and redistribute the biomechanical loads between the hip, knee, and ankle joints during barbell squats. These minor changes might affect musculoskeletal adaptations, essential for training and rehabilitation. However, the degree to which different heel heights affect people of different genders appears to be different, but in all participants, raising the heel reduces the dorsiflexion angle and muscle activation of the dorsiflexors, and increases the proportion of mechanical work performed by the ankle joint. Therefore, changes in heel height might have a potential effect on the technical movements of the barbell squat. Furthermore, regardless of the data results, athletes still report positive effects from elevated heel height, which might be psychological. Nevertheless, the results might vary between individuals with different training experiences and physical characteristics, and therefore, carefully choosing the shoes is necessary. In conclusion, for those with lower back and ankle injuries or mobility limitations, or those who wish to improve knee engagement during the barbell squat, we still recommend elevating the heel during the barbell squat to a height of no less than 1.5 cm.

## Figures and Tables

**Table 1:** Basic information of participants.

Index	Gender	
	Male	Female
n	10	10
Age (years)	24.00 ± 1.48	23.50 ± 1.12
Height (m)	1.75 ± 0.03	1.66 ± 0.02
Weight (kg)	79.00 ± 5.34	59.25 ± 3.96
Training age (years)	2.00 ± 0.71	2.75 ± 1.09

Participants’ height and weight tests were performed uniformly before the experiment.

**Table 2:** Joint angle, joint moment, and joint power.

Index		Male			Female			Average		
		0 cm	1.5 cm	3 cm	0 cm	1.5 cm	3 cm	0 cm	1.5 cm	3 cm
Hip	Max A (°)	−4.94 ± 9.37	−9.17 ± 4.53	−4.98 ± 13.44	−13.56 ± 5.11	−16.21 ± 4.67	−19.78 ± 4.23	−9.48 ± 8.54	−12.96 ± 5.79	−15.27 ± 10.67
	Min A (°)	−87.23 ± 9.45	−88.96 ± 9.27	−92.20 ± 12.67	−100.94 ± 8.21	−106.37 ± 7.24	−105.59 ± 13.34	−94.54 ± 11.15	−98.33 ± 11.97	−101.51 ± 14.51
	ROM (°)	82.29 ± 9.84	79.79 ± 7.60	87.23 ± 9.43	87.48 ± 8.44	90.16 ± 7.82	85.81 ± 12.89	85.06 ± 9.48	85.37 ± 9.29	86.24 ± 11.96
	Peak M (Nm/kg)	0.86 ± 0.12	0.81 ± 0.15	0.72 ± 0.02	0.68 ± 0.14	0.83 ± 0.20	0.79 ± 0.20	0.76 ± 0.16	0.82 ± 0.18	0.77 ± 0.17
	Peak CP (W/kg)	1.30 ± 0.34	1.28 ± 0.34	1.09 ± 0.24	0.90 ± 0.18	1.06 ± 0.22	1.00 ± 0.25	1.08 ± 0.33	1.17 ± 0.30	1.02 ± 0.25
	Peak EP (W/kg)	−0.86 ± 0.26	−0.98 ± 0.13	−0.97 ± 0.14	−0.75 ± 0.22	−0.93 ± 0.24	−0.97 ± 0.34	−0.80 ± 0.25	−0.94 ± 0.20	−0.97 ± 0.29
Knee	Max A (°)	−16.05 ± 7.04	−15.57 ± 3.33	−15.24 ± 5.97	−10.51 ± 7.25	−8.48 ± 4.79	−10.52 ± 5.82	−13.09 ± 7.67	−11.75 ± 5.48	−11.96 ± 6.25
	Min A (°)	−137.42 ± 3.97	−137.52 ± 3.92	−140.77 ± 4.60	−125.16 ± 10.51	−125.57 ± 5.84	−129.54 ± 7.80	−130.88 ± 10.18	−131.09 ± 7.81	−132.96 ± 8.68
	ROM (°)	121.38 ± 3.97	121.95 ± 3.26	125.53 ± 5.68	114.65 ± 16.89	117.09 ± 9.12	119.02 ± 11.81	117.79 ± 13.07	119.33 ± 7.45	121.00 ± 10.76
	Peak M (Nm/kg)	0.84 ± 0.08 ^c^	0.89 ± 0.07 ^c^	1.02 ± 0.10 ^ab^	0.86 ± 0.10	0.83 ± 0.09	0.90 ± 0.10	0.85 ± 0.09	0.86 ± 0.09	0.93 ± 0.11
	Peak CP (W/kg)	1.07 ± 0.23	1.08 ± 0.16	1.24 ± 0.23	1.13 ± 0.34	1.03 ± 0.24	1.06 ± 0.18	1.10 ± 0.29	1.06 ± 0.21	1.11 ± 0.21
	Peak EP (W/kg)	−1.41 ± 0.32 ^c^	−1.55 ± 0.34	−1.80 ± 0.38 ^a^	−1.08 ± 0.21	−0.99 ± 0.22	−1.21 ± 0.28	−1.23 ± 0.31	−1.25 ± 0.39	−1.39 ± 0.41
Ankle	Max A (°)	−1.66 ± 3.83 ^c^	0.29 ± 5.01	4.28 ± 3.01 ^a^	6.47 ± 2.91 ^bc^	9.33 ± 2.75 ^ac^	16.08 ± 1.11 ^ab^	2.67 ± 5.28 ^c^	5.16 ± 6.00 ^c^	12.49 ± 5.75 ^ab^
	Min A (°)	−36.43 ± 2.65	−36.14 ± 1.90	−34.80 ± 1.24	−31.81 ± 4.37 ^bc^	−26.12 ± 3.90 ^ac^	−21.09 ± 2.82 ^ab^	−33.97 ± 4.34 ^bc^	−30.74 ± 5.90 ^ac^	−25.27 ± 6.77 ^ab^
	ROM (°)	34.77 ± 4.96 ^c^	36.43 ± 3.50	39.08 ± 2.82 ^a^	38.28 ± 2.75 ^b^	35.45 ± 2.98 ^a^	37.17 ± 2.70	36.64 ± 4.31	35.90 ± 3.27 ^c^	37.75 ± 2.88 ^b^
	Peak M (Nm/kg)	0.73 ± 0.11	0.68 ± 0.05	0.70 ± 0.07	0.47 ± 0.05	0.47 ± 0.08	0.49 ± 0.08	0.59 ± 0.16	0.57 ± 0.12	0.56 ± 0.13
	Peak CP (W/kg)	0.53 ± 0.14	0.56 ± 0.09	0.66 ± 0.16	0.35 ± 0.09	0.34 ± 0.09	0.35 ± 0.08	0.44 ± 0.14	0.44 ± 0.14	0.44 ± 0.18
	Peak EP (W/kg)	−0.35 ± 0.10	−0.36 ± 0.10	−0.33 ± 0.07	−0.22 ± 0.04	−0.24 ± 0.06	−0.22 ± 0.07	−0.28 ± 0.10	−0.29 ± 0.10	−0.25 ± 0.08

When participants are not gender specific: ^a^ significant differences with 0 cm, ^b^ significant differences with 1.5 cm, ^c^ significant differences with 3 cm (A, angle; M, moment; CP, concentric power; EP, eccentric power). The joint angle and joint moment data were obtained from OpenSim, and the joint power data were processed using the joint angle and joint moment data.

**Table 3:** The relative proportion of work done by joints.

Index		Male			Female			Average		
		0 cm	1.5 cm	3 cm	0 cm	1.5 cm	3 cm	0 cm	1.5 cm	3 cm
Hip	NWR	0.32 ± 0.06 ^c^	0.31 ± 0.07 ^c^	0.25 ± 0.04 ^ab^	0.36 ± 0.08	0.44 ± 0.09	0.40 ± 0.12	0.34 ± 0.07	0.38 ± 0.10	0.35 ± 0.12
	PWR	0.38 ± 0.04 ^c^	0.36 ± 0.05	0.32 ± 0.04 ^a^	0.37 ± 0.06	0.44 ± 0.08	0.41 ± 0.10	0.38 ± 0.05	0.40 ± 0.08	0.38 ± 0.09
Knee	NWR	0.56 ± 0.08 ^c^	0.57 ± 0.10	0.63 ± 0.06 ^a^	0.54 ± 0.08	0.46 ± 0.10	0.52 ± 0.13	0.55 ± 0.08	0.51 ± 0.11	0.55 ± 0.13
	PWR	0.48 ± 0.06	0.50 ± 0.06	0.52 ± 0.05	0.50 ± 0.05	0.45 ± 0.08	0.48 ± 0.09	0.49 ± 0.06	0.47 ± 0.08	0.49 ± 0.08
Ankle	NWR	0.12 ± 0.04	0.12 ± 0.03	0.16 ± 0.05	0.09 ± 0.01 ^c^	0.09 ± 0.02 ^c^	0.15 ± 0.05 ^ab^	0.10 ± 0.03 ^c^	0.10 ± 0.03 ^c^	0.15 ± 0.05 ^ab^
	PWR	0.14 ± 0.03 ^c^	0.15 ± 0.02 ^c^	0.24 ± 0.03 ^ab^	0.13 ± 0.02 ^bc^	0.11 ± 0.01 ^ac^	0.20 ± 0.04 ^ab^	0.13 ± 0.03 ^c^	0.13 ± 0.02 ^c^	0.21 ± 0.04 ^ab^

When participants are not gender specific: ^a^ significant differences with 0 cm, ^b^ significant differences with 1.5 cm, ^c^ significant differences with 3 cm (NWR, negative work ratio and PWR, positive work ratio). The percentage of work done by the joints was obtained by processing the joint power data.

## Data Availability

The data that support the findings of this study are available on reasonable request from the corresponding author. The data are not publicly available due to privacy or ethical restrictions.
